# Signaling Pathways Regulating the Expression of the Glioblastoma Invasion Factor *TENM1*

**DOI:** 10.3390/biomedicines10051104

**Published:** 2022-05-10

**Authors:** María Carcelen, Carlos Velasquez, Verónica Vidal, Olga Gutiérrez, José L. Fernández-Luna

**Affiliations:** 1 Instituto de Investigación Vadecilla (IDIVAL), 39011 Santander, Spain; maria.carcelen93@gmail.com (M.C.); carlosjose.velasquez@scsalud.es (C.V.); veronicavisan@gmail.com (V.V.); molga.gutierrez@scsalud.es (O.G.); 2 Servicio de Neurocirugía, Hospital Univesitario Maqués de Valdecilla, 39008 Santander, Spain; 3 Servicio de Genética, Hospital Univesitario Maqués de Valdecilla, 39008 Santander, Spain

**Keywords:** glioblastoma, cell migration, tumor invasion, ODZ1, Stat3, HIF2α, hypoxia, promoter methylation

## Abstract

Glioblastoma (GBM) is one of the most aggressive cancers, with dismal prognosis despite continuous efforts to improve treatment. Poor prognosis is mostly due to the invasive nature of GBM. Thus, most research has focused on studying the molecular players involved in GBM cell migration and invasion of the surrounding parenchyma, trying to identify effective therapeutic targets against this lethal cancer. Our laboratory discovered the implication of TENM1, also known as ODZ1, in GBM cell migration in vitro and in tumor invasion using different in vivo models. Moreover, we investigated the microenvironmental stimuli that promote the expression of TENM1 in GBM cells and found that macrophage-secreted IL-6 and the extracellular matrix component fibronectin upregulated TENM1 through activation of Stat3. We also described that hypoxia, a common feature of GBM tumors, was able to induce TENM1 by both an epigenetic mechanism and a HIF2α-mediated transcriptional pathway. The fact that TENM1 is a convergence point for various cancer-related signaling pathways might give us a new therapeutic opportunity for GBM treatment. Here, we briefly review the findings described so far about the mechanisms that control the expression of the GBM invasion factor TENM1.

## 1. Introduction

Glioblastoma (GBM) is a grade IV glioma according to the WHO 2021 classification [1] responsible for 50% of malignant primary tumors of the brain [2]. Despite the multidisciplinary treatment based on surgery, chemo- and radiotherapy, patients have a median survival of 14–15 months from diagnosis [3]. 

The goal of surgery is the complete and safe resection of the tumor. Nevertheless, this is not possible in most patients due to the diffuse nature of GBM and its infiltration into the surrounding parenchyma implicated in functions such as language and movement. This feature is not exclusive to GBM but appears to be common to all gliomas regardless of grade [4]. Non-resected tumor cells can grow, migrate and invade, forming a new tumor, which is responsible for relapse in almost all GBM cases [5,6]. 

After resection, a major problem that patient faces is the resistance that most GBM tumors exhibit to current chemo- and radiotherapeutic strategies [7]. This resistance is largely due to the inter- and intratumoral heterogeneity of GBM. Within different areas of the same tumor, GBM cells have different mutations and phenotypes and variable epigenetic patterns that influence the response to treatment [8,9]. In addition, the diversity of the tumor microenvironment also contributes to developing treatment resistance mechanisms [10,11]. 

The process of tumor invasion has attracted great interest in the scientific community and the cellular and molecular players involved in this process have been extensively studied. At present, new pathways implicated in invasion are still being discovered. It is known that factors such as the composition of the extracellular matrix (ECM) and different phenotypes and epigenetic patterns of cells within the tumor influence the process of invasion [12].

Previous studies in our laboratory showed the implication of TENM1, Teneurin Transmembrane Protein 1, Teneurin-1, or ODZ1 (the symbol ODZ1 will be used here from now on) in the migration of GBM cells and invasion of the surrounding tissue [13]. Here, we briefly review the migratory and invasive capacity of GBM cells, the contribution of ODZ1 to these processes, the mechanisms that regulate ODZ1 expression, and the therapeutic approaches to control these pathways. 

## 2. Migration and Invasion of GBM

Migration and invasion are the results of the interplay of several molecular and cellular processes. Infiltration of the surrounding parenchyma involves two events that are usually linked, the migration of GBM cells and the invasion of the surrounding ECM. Briefly, GBM cells modify the adhesion to components of the ECM followed by enzymatic degradation (i.e., matrix metalloproteinases) of ECM, and promotion of cell motility by reorganization of the cytoskeleton [14]. An event that facilitates tumor invasion is that GBM cells communicate with endothelial cells and migrate along the vessels [15].

In a first step, migratory GBM cells modify their adherence to adjacent tumor tissue. This happens because expression changes occur in adhesion molecules such as cadherins, neuronal cell adhesion molecule (NCAM), and intercellular adhesion molecule 1 (ICAM1) [16,17]. Migrating cells will not lose adhesion to the central tumor, but will also modify their relationship with the ECM and other cells present in the microenvironment. Integrins act as regulators of cell adhesion to proteins of the ECM and are expressed by several cell types including endothelial and immune cells. It is also known that integrins activate different signaling pathways that promote the migration of GBM cells [17,18]. 

Simultaneously, GBM cells degrade the ECM by proteolytic enzymes such as metalloproteinases (MMP-2, MMP-9), promoting the invasion of the surrounding parenchyma. These enzymes have been extensively associated with the invasive capacity of the tumor. Moreover, features of the ECM, such as composition, geometry, and complexity, also modulate the invasive capacity of tumor cells. As an example, the presence of hyaluronic acid has been related to the upregulation of the NFκB pathway and in consequence to increased cellular migration [19,20]. 

During migration, remodeling of the cytoskeleton also occurs. Typically, migrating GBM cells become polarized and acquire a fibroblast-like shape. The plasma membrane extends to form a pseudopod with integrins to interact with the ECM. This interaction activates signaling pathways for migration leading to the recruitment of metalloproteinases and degradation of the protein matrix, which clears the pathway for migrating tumor cells [21]. The direction of cell migration follows the blood vessels, from which cells acquire oxygen and nutrients. 

The regulation of these processes is carried out by several molecular pathways, which represent both potential biomarkers of prognosis and potential therapeutic targets. Some of the best-known signaling pathways implicated in migration are PI3K-Akt, Wnt/β-catenin, RHO GTPases, Hedgehog and NFκB. Also, transcription factors such as STAT3 and C/EBPβ have been described as regulators of cell migration [13,17]. The majority of these signaling pathways show aberrant activation involved in processes related to the ECM. As an example, the PI3K signaling can be activated by several ligands, such as epidermal growth factor (EGF), and transforming growth factor beta (TGFβ). These processes lead to the activation of Akt kinases that promote the expression of genes related to cell growth and survival [17]. In GBM, as well as in other types of cancer, constitutive activation of this signaling pathway has been shown to take place by different mechanisms. In 50% of high-grade gliomas, a mutant variant of the EGF receptor (EGFRvIII) has been observed, leading to constitutive ligand-independent activation of the EGF receptor [22]. Also, in 40% of adult gliomas, mutations have been identified in the tumor suppressor phosphatase and tensin homolog (PTEN), the protein that regulates activation of the PI3K-Akt pathway [23]. 

It has been observed that voltage-dependent chloride and potassium channels are mainly used by GBM cells during migration. Additionally, oncogenic miRNAs, such as miR-21, miR-23a, miR-30a, miR-221, and miR-451, have been detected in GBM microvesicles and related to cell migration [14].

Some studies have shown that tumor clones responsible for recurrence conserve the genetic background of their progenitors, which could indicate that progenitor clones are in a dormant state waiting to repopulate the tumor [5,7]. Moreover, although the migratory capacity has classically been associated with differentiated cells, there is increasing scientific evidence for the migration of glioblastoma stem cells (GSCs), and therefore, for their role in tumor relapse.

## 3. ODZ1 and Its Role in Migration

Teneurins are a family of transmembrane proteins very conserved between species. The first teneurin was discovered in *Drosophila melanogaster* and lately in vertebrates [8], where four members of this family have been described (TENM1-TENM4). In vertebrates, teneurin genes are located on chromosome X and are mainly expressed during the development of the central nervous system, but they have also been involved in extra- and intracellular signaling and regulation of processes such as cell proliferation and adhesion [24,25,26]. 

This family of high molecular weight type II transmembrane proteins (about 300 KDa) consists of a big extracellular domain, with different conserved regions, a transmembrane domain, and an intracellular domain of 45 KDa. Intriguingly, the extracellular domain can be released [27]. Some studies have characterized the carboxyl-terminal end of the extracellular domain and have observed a structural similarity with the corticotropin-releasing factor and calcitonin peptides. Thus, it has been associated with a neuromodulatory activity [28,29]. This C-terminal end of the teneurin protein sequence has been named the Teneurin C-Terminal Associated Peptide (TCAP), and its functions are not clear yet. Some studies have described that it can generate changes in the cytoskeleton through the ERK signaling pathway [30] and interaction with latrophilins [31]. 

The intracellular region is linked to transcriptional regulation, although the target genes and mechanisms are not fully understood. In recent work, it was shown that the intracellular domain of TENM1 acts as a transcriptional regulator through HINT1, which binds to transcriptional factors and inhibits the expression of their target genes [27]. 

The evidence accumulated in the literature suggests the contribution of teneurins in tumorigenesis. Teneurin genes are subjected to typical changes in cancer-related genes such as somatic mutations or deregulated expression [24]. The aberrant expression of teneurins in different tumor contexts has been observed. For example, in liver, esophagus, and kidney tumors, teneurin expression is downregulated, while in brain tumors and lymphomas it has been observed an increase in teneurin levels [24,26]. Upregulation of ODZ1 (TENM1) expression has been associated with tumor progression and an invasive phenotype in papillary thyroid carcinoma [32,33]. Additionally, in differentiated thyroid cancer, ODZ1 has been proposed as a prognostic marker of disease-free survival, with increased expression in the tumor [34]. In a study of breast cancer, mutation of the ODZ1 gene has been related to the upregulation of a long non-coding RNA with prognostic value [35]. 

Our laboratory carried out the first study that described the implication of ODZ1 in the invasion capacity of GSCs [13]. We showed that ODZ1 promotes GSC migration and invasion of the surrounding environment, both in vitro and in vivo, by promoting actin cytoskeleton remodeling. ODZ1 expression was analyzed in GBM samples and showed increased expression in the invading area and in the periphery of neurospheres, consistent with the higher migratory capacity of these cells. The mechanism of action involved RhoA signaling, RhoA being a key player in the control of cytoskeleton dynamics and cell migration. Knocking down the expression of ODZ1 drastically reduced the invasive phenotype. 

Moreover, the upregulated expression of ODZ1 was predictive of poor outcomes both in patient and xenografts models.

With these previous data in mind, we aimed to describe the mechanisms that regulate ODZ1 gene expression. We hypothesized that cellular stressors in the tumor microenvironment might stimulate ODZ1 expression as part of the cellular response to unfavorable conditions. Next, we will review the mechanisms discovered so far. 

### 3.1. ODZ1 Expression Is Induced by Microenvironmental Signals through a Stat3-Dependent Transcriptional Pathway

The relationship between tumor cells and cells of the tumor microenvironment has been widely studied, and the bidirectional relationship between them is known. GBM cells try to modify their environment to make it more favorable for their growth and expansion [36]. GBM cells communicate with immune cells, among other cell types, in several ways, including the release of soluble factors in an attempt to modulate the behavior of immune cells [37]. 

Glioma-associated macrophages (GAMs) and microglia account for 30–50% of all cells in GBM tumors. A greater presence of microglia has been associated with early tumor stages, whereas more macrophages have been observed in advanced stages [37,38]. With the secretion of chemoattractants, such as fractalkine (CX3CL1) and colony-stimulating factor-1 (CSF-1), GBM cells recruit GAMs [39]. Furthermore, with the secretion of these and other chemokines, tumor cells redirect GAMs toward an immune-suppressive phenotype. 

However, it should be noted that, although two phenotypic states of macrophages have been classically described, M1 (pro-inflammatory) and M2 (anti-inflammatory), macrophages can be found in an intermediate degree of differentiation between both phenotypes [40]. Thus, GAMs under the influence of GBM cells may release both immune-suppressive factors, such as transforming growth factor β1 (TGF-β1) and interleukin 10 (IL-10), and also pro-inflammatory factors including interleukin 1 (IL-1) and tumor necrosis factor (TNF) [41]. 

At the same time, the cytokines and chemokines secreted by GAMs may promote tumor invasion. For instance, the release of TGF-β by GAMs upregulates integrins, induces MMP2 expression, and downregulates the inhibitor of metalloproteinases [42,43]. 

**Figure 1:**
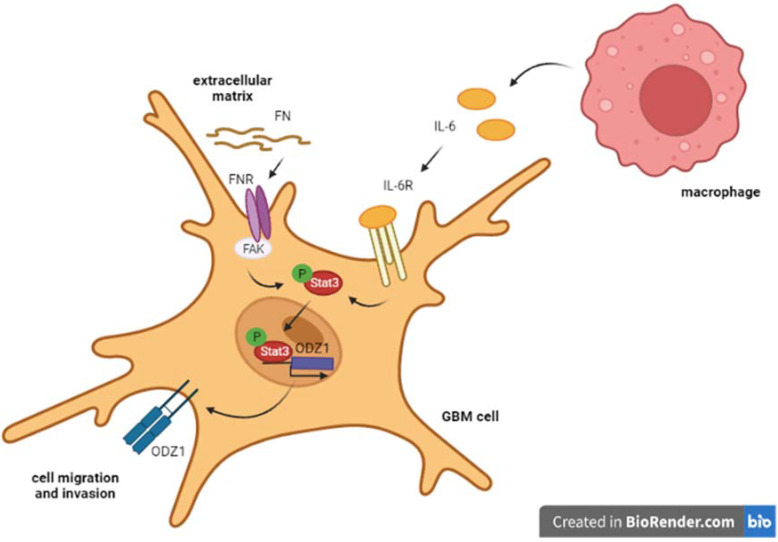
Schematic representation of the Stat3-dependent ODZ1 expression. IL-6 from activated macrophages and fibronectin of the ECM activated Stat3, promoting its phosphorylation. Activated Stat3 binds to the ODZ1 promoter and induces its expression [15].

With these data, we wondered if monocytic cells could induce the expression of ODZ1. We demonstrated that monocytic cells were in fact able to induce ODZ1 in GBM cell cultures through upregulation of the Stat3 signaling pathway [44]. More specifically, IL-6 secreted by monocytes activated the Stat3-mediated ODZ1 expression, which results in GBM cell migration (Figure 1). Overexpression of IL-6 has been linked to the induction of Stat3 phosphorylation and activation in GBM [45].

To confirm the regulation of ODZ1 expression by a Stat3 signaling pathway we previously described three strategies that include blockade of the IL-6 receptor, inhibition of Jak kinases, and knockdown of Stat3. In all cases the expression of ODZ1 was reduced and consequently, cell migration decreased [46]. Furthermore, the ODZ1 gene promoter contained a consensus Stat site, and mutagenesis of this site prevented activation of the promoter. 

As we have already mentioned, components of the ECM are also important for the migration process. In line with this, fibronectin, which is a main component of the GBM ECM, has been shown to trigger the Stat3 pathway and was found to be another inducer of the Stat3-mediated ODZ1 expression in GBM cells [46].

Stat3 has been associated with immunological functions but also with growth in several tumors, including gliomas [46]. In another study, GBM patients were stratified into Stat3-high and Stat3-low activation, and the inhibition of Stat3 in Stat3-high patients reduced tumorigenicity [47]. Moreover, increased levels of IL-6 have been observed in the cerebrospinal fluid of GBM patients [48]. 

One of the most common mutations in GBM, the deletion of exons 2–7 of the EGFR gene, called EGFRvIII, has been shown to activate Stat3 and Stat5 transcriptional pathways contributing to cell cycle progression and preventing apoptosis of glioma cells [49].

Consistent with these results, other studies showed activation of Stat3 in GBM cells through different mechanisms mediated by Guanylate-binding protein 2 (GBP2) [50], the transcription factor NFκB [51], and the orphan receptor of the TNF receptor superfamily TROY [52]. Activation of the Stat3 signaling pathway leads to the nuclear translocation of Stat3 where it binds to promoter regions of target genes such as Myc, Bcl-2, cyclin D1, VEGF, and HIF-1α, some of which have been related to cell migration and tumor invasion [49]. 

Based on previous findings, several inhibitors of the IL6-Stat3 pathway have been developed (embelin, quercetin), which could also reduce the expression of ODZ1 in GBM [53,54]. Another therapeutic strategy, that would also impact ODZ1 expression indirectly, could be the use of chimeric antigen receptor macrophages (CAR-M), which are modified to express a pro-inflammatory phenotype [55]. 

### 3.2. ODZ1 Expression Is Induced under Hypoxic Conditions

The rapid growth of the tumor generates regions with strongly disordered angiogenesis, leaving avascular tissue with decreased oxygen availability known as hypoxic regions. These regions have been correlated with malignancy, poor prognosis, and resistance to chemotherapy. In addition, low oxygen levels appear to reduce the effectiveness of radiation therapy. On the other hand, tumor cells reprogramme their transcriptional activity facilitating adaptation to a new environment, which ultimately favors cell survival, motility, and angiogenesis [56]. Hypoxia has been associated with several aspects of the biology of GSCs, such as generation, maintenance, and invasiveness [57,58].

Tumor cells respond to the hypoxic microenvironment through changes in gene expression. It is known that hypoxia stimulates signaling pathways such as those triggered by PI3K/Akt and tyrosine kinase receptors [59,60,61]. With this in mind, our group evaluated whether ODZ1 could also be upregulated under hypoxic conditions, as is the case of other proteins involved in migration. It was found that hypoxia promoted ODZ1 expression by two different mechanisms: activation of hypoxia-inducible factors (HIFs) and epigenetic changes of the ODZ1 promoter.

#### 3.2.1. HIF Signaling

The main and best-known elements that mediate the response of cells to hypoxia are the hypoxia-inducible factors (HIFs). HIFs are heterodimeric proteins composed of two subunits (α and β) that are degraded under normal conditions, but that in hypoxic environments are stabilized and activated, promoting the expression of a number of target proteins [56,62]. Three isoforms of HIF have been described, being HIF1α and HIF2α the most well studied. These proteins are highly homologous and have common features but also unique tissue distribution and different target genes [63,64]. Common target genes of HIF1α and HIF2α include vascular endothelial growth factor (VEGF), and glucose transporter 1 (GLUT-1), among others. Individually, HIF1α has been associated with the metabolic reprogramming of glycolysis, the expression of genes involved in apoptotic cell death, and the suppression of anti-tumor immune effects [65], while HIF2α has been associated with the maintenance of GSCs and an immunosuppressive phenotype in tumor-associated macrophages [62,66]. 

In the last decades, GSCs have acquired great relevance and their fundamental role in the pathogenesis and recurrence of GBM is widely accepted [12,67]. Given the described relationship between the hypoxic regions of the tumor with the maintenance of GSCs and the invasive properties [68,69], we hypothesized that ODZ1 could also be regulated by HIFs under hypoxia conditions in GSCs. 

**Figure 2:**
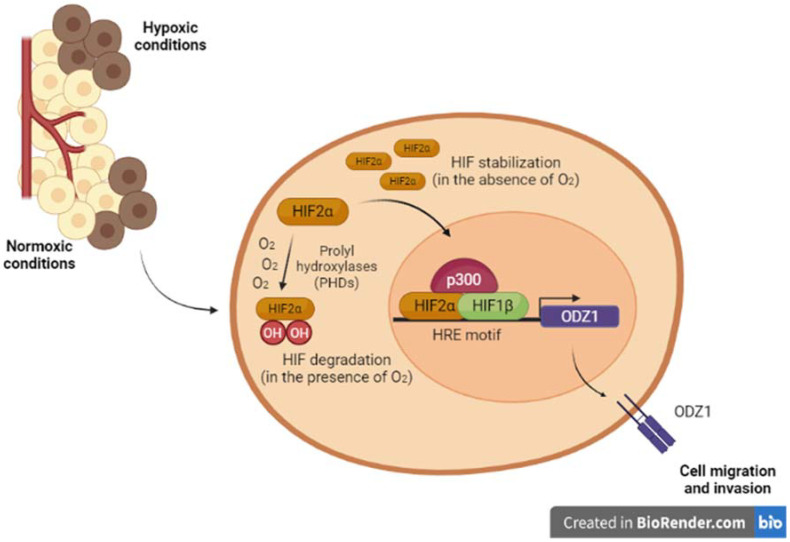
HIF2α-ODZ1 gene expression pathway. HIFs are heterodimeric proteins, composed of alpha (α) and beta (β) subunits. In normal conditions, HIFα is subjected to ubiquitination and proteasomal degradation. However, under hypoxia, HIF is stabilized and translocated to the nucleus. In the nucleus, HIFα (HIF1α, HIF2α, or HIF3α) dimerizes with HIF1β and the heterodimer binds to hypoxia-response element (HRE), a specific sequence (RCGTG) located within the promoter of target genes, including ODZ1, inducing their expression [70].

We cultured three primary GSC cell lines under hypoxia or treated with HIF regulators DMOG, which is a HIF protein stabilizer, and Chetomin, a HIF signaling disrupter, and observed the regulation of ODZ1 expression [70]. Cultures under hypoxia or treated with DMOG, that mimics hypoxia, showed an upregulated expression of ODZ1. We also identified the presence of a HIF binding site (HRE, hypoxia-response element) in the promoter of ODZ1. Luciferase gene reporter assays showed that GSC cultures treated with DMOG activated the ODZ1 promoter [70]. Furthermore, downregulation of HIF2α with specific siRNAs reduced the expression of ODZ1. Taken together, our data showed the transcriptional regulation of ODZ1 by HIF2α in GSCs under hypoxic conditions (Figure 2).

HIFs not only favor the maintenance of GSCs and the generation of an immunosuppressive state in the tumor microenvironment [58] but also promote the invasiveness of GBM cells. To this end, the enzyme PLOD2 (procollagen-lysine 2-oxoglutarate 5-dioxygenase 2), which regulates collagen crosslinking, has been shown to have a HIF-dependent expression [71]. Other HIF-regulated genes implicated in GBM cell migration are ZEB1 (zinc finger E-box-binding homeobox 1), TWIST1 (Twist-related protein 1), and chemokines such as CXCR4 and its ligand CXCL12, and CCR5 and its ligand CCL4 [71,72], and now, we include ODZ1 as a relevant hypoxia-inducible factor in GSCs. 

The contribution of HIF isoforms to the poor prognosis of GBM is not entirely clear. In a recent study, researchers showed that knocking out HIF1α or HIF2α individually had no significant changes in cell proliferation and cell cycle progression in acute hypoxia situations. However, knocking out HIF1α and HIF2α simultaneously had significant changes in cell proliferation, promoting differentiation and chemosensitivity. They observed that HIF1α and HIF2α regulate each other in negative feedback [61]. Another study correlated HIF2α, but not HIF1α, with poor prognosis, suggesting a predominant role of HIF2α in GBM [69]. 

In this work, we analyzed the relationship between ODZ1 and HIF2α. It would be interesting to study the contribution of different hypoxia-activated pathways to the overall migration capacity of GBM cells and GSCs. Finding common points in these signaling pathways may provide us with more effective therapeutic targets. 

#### 3.2.2. Epigenetic Regulation

Besides HIFs, epigenetic modifications also contribute to the cellular response to hypoxia. Epigenetic changes allow cells to adapt their transcriptome to specific conditions in a reversible and rapid way. The global hypomethylation in tumor cells, with specific hypermethylation in the promoters of tumor-suppressor genes, is widely accepted [73,74]. 

Under hypoxia, tumor cells are subjected to changes in DNA methylation and histone modification to improve their survival and progression in this stressful environment. An example of this is the hypermethylation of CpG islands in the Stat6 promoter by DNA methyl transferases (DNMTs) under hypoxia. This hypermethylation induces the mTOR signaling activation and the accumulation of HIF1α, which facilitates the survival of glioma cells [75]. These results are in line with the hypermethylation of promoters of tumor-suppressor genes by downregulation of ten eleven translocation proteins (TETs) activity under hypoxia [76]. 

Our group has recently discovered the regulation of ODZ1 by an epigenetic mechanism in hypoxic conditions [77]. In tissue samples from GBM patients, ODZ1 expression was upregulated in hypoxic areas compared with non-hypoxic areas, which are delimited by using a hypoxia biomarker. Additionally, primary GBM cultures were incubated under hypoxic conditions and both the expression of ODZ1 and cell migration were increased. Conversely, silencing of ODZ1 reduced the hypoxia-promoted migration. 

CpG islands appear in about 60% of human promoters repressing their activation [78]. Analysis of the TCGA database comparing the methylation status of CpG sites in the ODZ1 gene in GBM tissue and normal brain revealed two CpG sites hypomethylated in GBM and only one of these sites (cg24761295), closed to the transcription start site, was confirmed to be hypomethylated in a prospective study with tumor specimens from 10 GBM patients [77]. 

To further confirm this finding, CpG sites in the ODZ1 promoter were enzymatically methylated and a reporter plasmid containing the methylated promoter was transfected into GBM cells. It was found that the promoter activity was drastically reduced under hypoxia when CpG sites were methylated and site-directed mutagenesis revealed that the cg24761295 site was the main one responsible for this reduction. 

**Figure 3:**
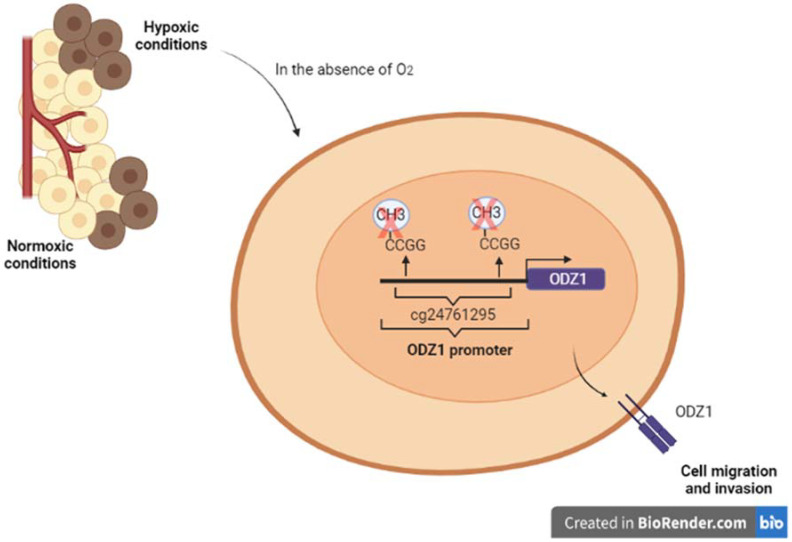
Hypoxia regulates the expression of ODZ1 by hypomethylation of a CpG site in the ODZ1 promoter. In samples of GBM patients, hypoxic and normoxic regions were identified by immunohistochemistry and the methylation status of CpG sites within the ODZ1 promoter was analyzed. Hypoxic GBM cultures showed a hypomethylation status of a CpG site (cg24761295). This hypomethylation facilitates ODZ1 expression, and in consequence, cell migration [21].

In summary, the results showed the role of ODZ1 promoter methylation status in the ODZ1 expression under hypoxia in GBM cells (Figure 3) [77]. 

Other researchers have studied teneurins expression under different epigenetic changes in cancer models and the results are controversial. A likely explanation is that it depends on the teneurin member analyzed, the epigenetic stimulus used, and the type of tumor tissue. For example, TENM2 and TENM4 methylation was not affected in breast and ovarian cancer cells, however, the TENM3 promoter was found to be hypermethylated in early breast lesions [24]. 

Clinical trials have been carried out with several molecules such as panobinostat and romidepsin, trying to modify the epigenetics of GBM cells. However, the results have not been good enough so far. A clinical trial is currently underway testing folic acid, which may have the potential to restore abnormal DNA methylation, as an adjunct to temozolomide and radiation in GBM [79]. 

Although the first epigenetic mechanism that regulates ODZ1 expression has been discovered, other regulation mechanisms including histone modification and miRNAs might also contribute to the control of ODZ1 expression in response to hypoxia or other stress stimuli. In addition, it is also likely that a hypoxic microenvironment activates the Stat3 signaling pathway, as it has been shown in other systems [80], to induce ODZ1. 

## 4. ODZ1 as a Potential Therapeutic Target

Basic science constantly proposes potential therapeutic targets, but intense translational and clinical research is needed to incorporate them into clinical practice. Many clinical trials are being conducted for treating patients with GBM. One of the phase I trials is based on the use of the Jak2 inhibitor WP1066, which blocks signaling through Stat3, in patients with recurrent GBM (ClinicalTrials.gov Identifier: NCT01904123). Stat3 is one of the transcription factors shown to promote the expression of ODZ1 in response to IL-6 and fibronectin. Other signals that may activate Stat3 include EGF. Interestingly, the EGF receptor (EGFR) is one of the targets most commonly addressed in clinical trials for GBM. In line with this, a recent study showed that constitutively active EGFR correlated with overexpression of ODZ1 [81]. Whether this correlation is linked through Stat3 is still uncertain. Another phase II trial uses Tocilizumab, a monoclonal antibody that avoids binding of IL-6 to its cognate receptor, in recurrent GBM patients (NCT04729959). We showed that treatment of GBM cells with Tocilizumab was able to reduce the expression of ODZ1 in response to IL-6 and decrease the migratory capacity of these tumor cells. It would be of interest to analyze the levels of ODZ1 in the tumors after treatment. However, in the context of this clinical trial, one of the drawbacks of this approach is that surgery for recurrent GBM is associated with increased postoperative morbidity which precludes a surgical procedure in many patients. Another transcription factor that we have shown to upregulate ODZ1 is HIF2α, which becomes activated under hypoxic conditions, a characteristic of GBM that limits tumor response to chemotherapy. At least three different HIF2α inhibitors, PTP385 in patients with recurrent GBM (NCT03216499), MBM-02 (NCT04874506) for newly diagnosed GBM, and Belzutifan in patients with advanced tumors (NCT02974738) have been included in currently active or completed clinical trials. A prospective study of tumor tissue specimens from newly diagnosed GBM patients treated with HIF2α inhibitors would shed light on the potential role of the HIF2α-ODZ1 cell migration-associated transcriptional pathway in disease progression.

All pathways addressed by the above-mentioned clinical trials have multiple downstream targets. However, we have shown that ODZ1 is a common effector protein in all of them that may represent an advantage for GBM cells to migrate out of the tumor invading the surrounding parenchyma. We have seen that the infiltrative growth behavior of GBM is a major therapeutic challenge. If a number of key signaling pathways in GBM cells converge in the migration factor ODZ1, we can hypothesize that targeting ODZ1 could be a potentially effective therapeutic strategy to avoid tumor recurrence in GBM patients.

We have demonstrated the relationship between ODZ1 expression and survival in GBM patients [13]. The number of ODZ1-positive cells inversely correlated with both overall survival and disease-free survival when 122 samples from GBM patients were studied. Taking disease-free survival as the time from the resection to the first radiological recurrence, this parameter increased by 45% in patients with lower expression of ODZ1 compared to those with higher expression, and the overall survival increased by 24% [13]. 

These results are in line with data from the Repository for Molecular Brain Neoplasia Database (Rembrandt), which described a significantly higher expression of ODZ1 in grade IV gliomas (with worse prognosis) than in grade III gliomas. 

These data are consistent with results obtained from in vitro and in vivo models showing the relevance of ODZ1 in promoting invasive capabilities in GBM cells [13]. Mice xenografted with ODZ1-overexpressing GBM cells had larger tumors and shorter survival than their control counterparts. Moreover, ODZ1 levels controlled the capacity of GBM cells to propagate through the surrounding tissue in chicken embryos. 

Taking into consideration the functional studies, the in vivo models, and the regulatory pathways that modulate the expression of ODZ1, which are key pathways in the pathogenesis and/or development of GBM, there seems to be some good evidence to postulate ODZ1 as a potential therapeutic target. 

## 5. Conclusions

In conclusion, despite the current therapies to fight GBM, new tools are needed to combat recurrence, increase progression-free survival and, consequently, increase life expectancy. Our group has shown the role of ODZ1 in GBM cell migration and invasion of surrounding tissue and the transcriptional regulation of ODZ1 by several key pathways involved in GBM pathogenesis. The fact that ODZ1 expression is a convergence point for several pathogenic signaling pathways gives us an idea of its importance, and also makes it an interesting potential therapeutic target since it would attack tumor invasion promoted by different stimuli.
